# Gas Phase Transformations in Carbon-11 Chemistry

**DOI:** 10.3390/ijms25021167

**Published:** 2024-01-18

**Authors:** Shuiyu Lu, Sanjay Telu, Fabrice G. Siméon, Lisheng Cai, Victor W. Pike

**Affiliations:** Molecular Imaging Branch, National Institute of Mental Health, National Institutes of Health, Building 10, Rm B3C346, 10 Center Drive, Bethesda, MD 20892-1003, USA; shuiyu.lu@mail.nih.gov (S.L.); sanjay.telu@nih.gov (S.T.); fabrice.simeon@nih.gov (F.G.S.); lishengcai@mail.nih.gov (L.C.)

**Keywords:** carbon-11, gas phase, catalysts, on-line processes, radiotracer, radiochemistry, PET

## Abstract

The short-lived positron-emitter carbon-11 (*t*_1/2_ = 20.4 min; β^+^, 99.8%) is prominent for labeling tracers for use in biomedical research with positron emission tomography (PET). Carbon-11 is produced for this purpose with a cyclotron, nowadays almost exclusively by the ^14^N(p,α)^11^C nuclear reaction, either on nitrogen containing a low concentration of oxygen (0.1–0.5%) or hydrogen (~5%) to produce [^11^C]carbon dioxide or [^11^C]methane, respectively. These primary radioactive products can be produced in high yields and with high molar activities. However, only [^11^C]carbon dioxide has some utility for directly labeling PET tracers. Primary products are required to be converted rapidly and efficiently into secondary labeling synthons to provide versatile radiochemistry for labeling diverse tracer chemotypes at molecular positions of choice. This review surveys known gas phase transformations of carbon-11 and summarizes the important roles that many of these transformations now play for producing a broad range of labeling synthons in carbon-11 chemistry.

## 1. Introduction

**Scheme 1:**
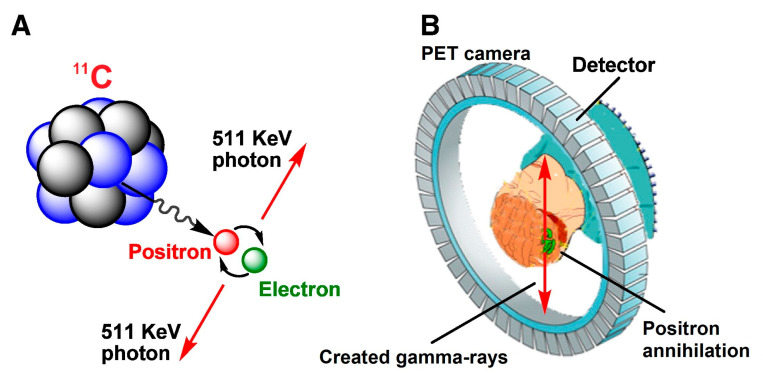
Decay of carbon-11 (**A**) and principle of PET (**B**). Reprinted and modified from Li and Conti, 2010 [3], with permission from Elsevier.

The short-lived positron-emitter carbon-11 (*t*_1/2_ = 20.4 min) has prominent application for labeling organic tracers that are used in biomedical research, drug development, and disease diagnosis with the molecular imaging technique of positron emission tomography (PET) [1]. Carbon-11 decays almost purely by positron emission (β^+^, 99.8%) with the remainder by electron capture, each producing stable boron-11. The emitted positron has average and maximum energies of 0.3856 and 0.980 MeV, respectively, providing it a short range in dense tissue before being annihilated by combining with an electron. Positron annihilation results in emission of a pair of antiparallel photons, each with an energy of 511 keV. The photons can readily escape from deep tissue. Their coincident detection provides the basis for locating the administered tracer (or any arising radiometabolite) responsible for the positron emission within a living subject, either animal or human (Scheme 1). Nowadays, there are highly sophisticated ‘PET cameras’ that can record the distribution of positron emissions with time in the whole animal or human body or in major organs such as brain or heart. PET scans with a physical resolution of about a millimeter can be achieved every few seconds or minutes [2]. The biomedical information gained depends on the design of the tracer. Most tracers are designed to provide information on the distributions of a particular low-density protein (e.g., neuroreceptor, transporter, enzyme, or pathological plaque), or on the protein interaction with either an endogenous compound (e.g., neurotransmitter) or a known or experimental drug. PET is highly sensitive, being able to detect proteins at sub-nanomolar concentrations. Because of its short half-life, carbon-11 must be rapidly produced and transformed into a PET tracer for time of need. This review surveys gas phase transformations of carbon-11 and highlights the especially strong enabling role that they now play in achieving the aims of rapid, efficient, and reliable PET tracer production.

## 2. Carbon-11 Production

Carbon-11 may be produced for PET by many different cyclotron-promoted nuclear reactions [4,5]. Early methods used irradiations of solid targets, such as the ^10^B(d,n)^11^C, ^11^B(d,2n)^11^C, and ^11^B(p,n)^11^C reactions on boron trioxide [6]. Nowadays, however, carbon-11 is produced almost exclusively by the much higher yielding [7,8] and more easily manageable ^14^N(p,α)^11^C nuclear reaction on high pressure nitrogen gas [9]. Typically, the nitrogen has an initial pressure of about 225 psi, and irradiations are performed with moderate to high beam currents (typically 40–150 μA) of moderate energy protons (~16.5 MeV). Many compact biomedical cyclotrons are commercially available for this purpose [10,11]. For example, in our laboratory, over 3 curies of carbon-11 can be produced from a 40-minute irradiation of a nitrogen–oxygen mixture with a 45 μA beam of 16.5 MeV protons from a PETtrace cyclotron. By contrast, with this scale of carbon-11 production, only 10 to 20 mCi of a tracer is typically required to be administered to a human subject for a PET experiment.

**Scheme 2:**
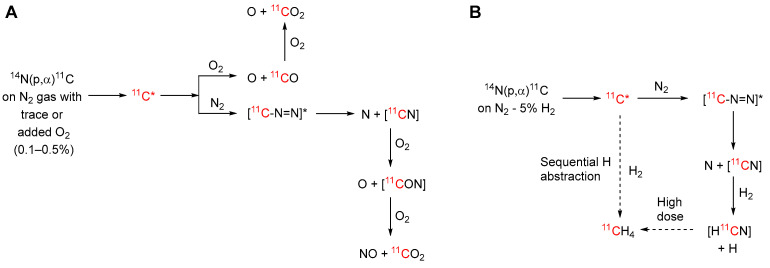
Processes leading to [^11^C]carbon dioxide and [^11^C]methane by proton irradiation of nitrogen gas with low level oxygen (**A**) or ~5% hydrogen (**B**), respectively. * Indicates hot atom or energetic species.

The chemical form of the retrieved carbon-11 depends on both cyclotron target gas composition and irradiation dose (Scheme 2) [9,12]. Initially, for the proton irradiation of nitrogen, [^11^C]cyano radicals and [^11^C]carbon monoxide are formed by recoil reactions of nucleogenic ^11^C atoms with nitrogen and traces of oxygen, respectively. Even at a low radiation dose (10^−3^ eV molecule^−1^s^−1^), a trace of oxygen (1–10 ppm) radiolytically oxidizes the [^11^C]cyano radical to [^11^C]carbon dioxide. At high irradiation doses, [^11^C]carbon monoxide also oxidizes radiolytically to [^11^C]carbon dioxide, leaving only a trace of [^11^C]carbon monoxide. Thus, a relatively intense proton irradiation of high-purity nitrogen that contains trace oxygen is an effective means for producing [^11^C]carbon dioxide in high radiochemical purity and high activity (Scheme 2A). The product is free of chemical impurities, provided that only a low concentration of oxygen (1–10 ppm) is present. Normally, however, the cyclotron target body is made of aluminum which may consume traces of oxygen and lead to diminishing radioactivity recovery over many successive irradiations. Therefore, oxygen (0.1–0.5% *v*/*v*) is normally added to the nitrogen target gas to ensure reliable and consistently high recovery of carbon-11 radioactivity. However, the higher concentrations of oxygen generate potentially troublesome nitrogen oxides by radiolysis. These can be removed by on-line passage of the recovered irradiated gas into a trap filled with a mixture of chromium trioxide, copper sulfate, and 2 M sulfuric acid that has been dried onto a silica gel support [13]. Alternatively, the recovered [^11^C]carbon dioxide can be concentrated in a trap composed of a small coil of stainless steel tube immersed in a cryogen, either liquid nitrogen or liquid argon. Liquid argon is preferred because its higher temperature (−185 °C) avoids the co-trapping of nitrogen (bp, −196 °C) and oxygen (bp, −183 °C). Nitrogen trapped by liquid nitrogen cooling may cause a rapid and poorly controllable gas expansion upon the release of [^11^C]carbon dioxide at room temperature into a flowing inert gas stream. Alternatively, [^11^C]carbon dioxide can be trapped without any of the added oxygen on molecular sieves (3, 4, or 5 Å) at room temperature and then released in the concentrated form by a flush with an inert gas at higher temperature [14]. This method is overall efficient and reliable, and therefore widely used.

If hydrogen (~5% *v*/*v*) is present in the irradiated nitrogen target gas, nucleogenic carbon-11 atoms either react with nitrogen to generate [^11^C]cyano radicals, which then react with hydrogen to form [^11^C]hydrogen cyanide, or they sequentially abstract hydrogen to produce [^11^C]methane (Scheme 2B). At a low dose rate (10^−4^ eV molecule^−1^ s^−1^), a significant proportion of the radioactivity is recovered as [^11^C]hydrogen cyanide. However, at a high dose rate (>0.1 eV molecule^−1^ s^−1^), the radiolytic reduction of [^11^C]hydrogen cyanide becomes very significant, and nearly all the carbon-11 (>95%) is retrieved as [^11^C]methane. Thus, high activities of [^11^C]methane can be produced. [^11^C]Methane is readily recovered and concentrated from irradiated target gas by passage into a Porapak N trap at room temperature or a Porapak Q trap at −186 °C [15]. [^11^C]methane can then be released at a higher temperature.

The experience of many laboratories is that the recovered activities of [^11^C]methane are substantially lower than the recovered yields of [^11^C]carbon dioxide for the same energy and beam current in high-intensity production irradiations. For example, Buckley et al. [16]. reported that the recovery of [^11^C]methane was only 65% of that for [^11^C]carbon dioxide from the same irradiation conditions on the same target chamber. However, some laboratories report appreciably higher molar activity for [^11^C]methane than [^11^C]carbon dioxide [15,17].

The potential to produce predominantly [^11^C]hydrogen cyanide by the proton irradiation of nitrogen–hydrogen gas mixtures has been well explored but has not delivered a really practical method for high-level production. For example, a yield of 0.67 curies of [^11^C]hydrogen cyanide has been produced from an irradiation of a flowing nitrogen–(1%) hydrogen mixture (60 psi) in a heated (200 °C) quartz-lined target with 30 μA of 15 MeV protons for 30 to 45 min [18,19]. However, this yield represents only about 20% of that expected for carbon-11 from such an irradiation. 

In PET imaging experiments on animals and human subjects, the mass of administered tracer must be limited to avoid possible toxicity and to comply with the tracer principle, which is to avoid perturbation of the biochemical system being studied. In this regard, an extremely important consideration in the production of carbon-11 is the molar activity (*A*_m_) of the chemical species that is produced. The molar activity is defined as the ratio of radioactivity (e.g., in Ci or GBq) to the total mass of all isotopologues (e.g., in μmol) of the chemical species in question at a specific time, such as the end of the radionuclide production (ERP). *A*_m_ values decrease with the decay of the radionuclide. For an ^11^C-labeled product, the isotopologues to be considered are the corresponding natural abundance ^12^C and ^13^C isotopologues. As mentioned above, cyclotron irradiation may produce a few curies of carbon-11. One curie of carbon-11 corresponds to about 0.1 nmol, an extremely small amount of substance with a theoretical molar activity of 9200 Ci/μmol. However, cyclotron irradiations typically produce carbon-11 with much lower molar activities of 20 to 100 Ci/μmol. This is because the sources of trace non-radioactive isotopologues, known as carrier, may enter the cyclotron target or the radioactive product recovery system. For example, during [^11^C]carbon dioxide production, carbon dioxide may be produced from organic materials during irradiation, or a trace of atmospheric carbon dioxide may contaminate the target or product recovery apparatus. Therefore, scrupulous measures are required to minimize the ingress or production of trace carrier [20,21]. This includes, for example, using ultra-pure nitrogen, as free as possible of any hydrocarbons and carbon dioxide, as the target gas. The application of careful measures to eliminate potential sources of carrier in post-irradiation chemical processing can lead to good conservation of molar activity [22,23]. Labeled products are described as no-carrier-added (NCA) where such measures are reasonably taken. If carrier is deliberately added, they are described as carrier-added (CA). In the remainder of this review, the discussed products are NCA, unless mentioned as being CA. Herein, cited *A*_m_ values are for the end of radiosynthesis (EOS), and radiochemical yields are decay-corrected, unless otherwise stated. *A*_m_ values and yields are reported as a mean ± SD, unless a single value is otherwise stated. Table 1 is provided for the ease of locating the discussion on each ^11^C-labeled compound produced by a gas phase transformation.

An ability to produce tracers for PET imaging depends on being able to convert a primary cyclotron-produced product, either [^11^C]carbon dioxide or [^11^C]methane, into the tracer by rapid and high-yielding post-irradiation chemical means. In practice, only two or three physical half-lives can be allowed for a full tracer production, including the separation and formulation of tracer for intravenous administration. Because of its reactivity, [^11^C]carbon dioxide has some direct but limited utility (e.g., for ^11^C-carboxylation reactions). However, [^11^C]methane must be transformed into some other labeling agent to be useful. Similarly, the chemical transformations of [^11^C]carbon dioxide can produce more useful labeling agents. 

Whereas the preponderance of carbon-11 chemistry is performed in solution, methods for performing ^11^C-chemistry in the gas phase are highly attractive. They can often be performed on-line and very rapidly in a flow of suitably inert carrier gas (e.g., nitrogen or helium), and they allow easy product isolation, often in a solvent of choice. Catalysts and reactants can often be used repeatedly, and the apparatus can be readily automated for the protection of personnel from radioactivity. We now discuss known and important post-irradiation gas phase transformation methods in carbon-11 chemistry, indicating how they have been successful. Opportunities for improvement and expansion will also become apparent. 

## 3. Conversions of Cyclotron-Produced ^11^C-Labeled Materials

### 3.1. Conversions of [^11^C]Carbon Dioxide

#### 3.1.1. Into [^11^C]Methane

**Scheme 3:**
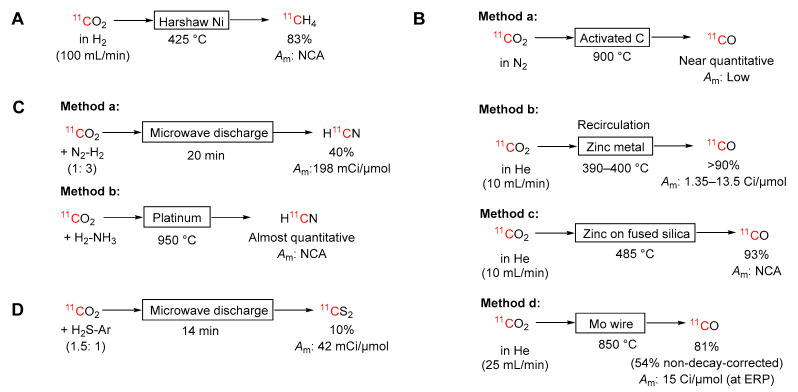
Conversions of [^11^C]carbon dioxide into other ^11^C-labeling agents: (**A**) [^11^C]methane; (**B**) [^11^C]carbon monoxide; (**C**) [^11^C]hydrogen cyanide; and (**D**) [^11^C]carbon disulfide.

[^11^C]Methane is a useful precursor for other labeling agents, as discussed below (Section 3.2). Because [^11^C]carbon dioxide can be obtained in higher activity than [^11^C]methane, it can be advantageous to convert cyclotron-produced [^11^C]carbon dioxide into [^11^C]methane with an efficient on-line process. This can be achieved simply by passing [^11^C]carbon dioxide with hydrogen over a nickel catalyst at temperatures between 370 and 450 °C (Scheme 3A) [9,24,25,26,27,28]. Various physical forms of the nickel catalyst have been used. Kniess et al. [28] compared Harshaw nickel, Shimalite nickel, nickel on silica–alumina (65 wt%), and nanosize (90 nm) pure (99.99%) nickel. High radiochemical yields (83%) were obtained by the use of Harshaw nickel at 425 °C. In our laboratory, this method routinely produces very high molar activity for derived PET tracers (40 ± 16 Ci/μmol at ERP).

#### 3.1.2. Into [^11^C]Carbon Monoxide

[^11^C]Carbon monoxide has gained major interest for labeling carbonyl groups in compounds such as ketones, amides, ureas, and carboxylic acids through palladium- and other transition metal-mediated processes. These methods have been reviewed extensively [29,30,31]. Although solution-phase methods for producing [^11^C]carbon monoxide are now known, the on-line gas phase reduction of cyclotron-produced [^11^C]carbon dioxide is still a popular approach because of its simplicity and convenience. Several methods have been described (Scheme 3B).

The earliest gas phase method for producing [^11^C]carbon monoxide was based on the reduction of [^11^C]carbon dioxide over activated charcoal at 800–900 °C (Method a, Scheme 3B) [32,33]. This method produces near-quantitative yield but results in low molar activity, presumably because of unavoidable low-level carbon oxidation. This method is virtually obsolete for radiosynthesis applications because PET tracers usually need to be produced at high NCA molar activity.

[^11^C]Carbon dioxide can be reduced efficiently to [^11^C]carbon monoxide by passage over zinc metal heated between 390 and 400 °C (Method b, Scheme 3B) [34,35]. A single pass can produce high yield (~70%) [35]. Almost quantitative yield has been achieved by the preconcentration and recirculation of [^11^C]carbon dioxide over the heated zinc [36]. High molar activities (1.35 to 13.5 Ci/μmol) are repetitively attainable [36]. However, success with this method depends very much on the quality of the zinc. Yields may become irreproducible if oxides form on the zinc over successive heating cycles. Performance may also vary unpredictably from one batch of zinc to another. Furthermore, the required temperature is not far below the melting point of zinc (420 °C). Therefore, the overheating of the zinc column must be carefully avoided.

The use of solid-supported zinc has been proposed for improving the gas phase production of [^11^C]carbon monoxide [37]. Molecular sieves, fused silica (prepared from silica gel), and molybdenum have been investigated as solid supports with fused silica proving to be preferred. The use of a column of zinc supported on fused silica at 485 °C produced impressive yields (93 ± 3%*, n* = 20) (Method c, Scheme 3B). This approach overcame the main limitations of fast deactivation and potential zinc metal melting that are experienced with the traditional heated zinc column. 

Molybdenum wire heated to 850 °C within a quartz tube is an alternative reductant to zinc [38]. [^11^C]Carbon monoxide is obtained in up to 81% yield and with high molar activity (up to 15 Ci/μmol) (Method d, Scheme 3B). The process can be completed within 15 min and can be operated for a large number of runs without the need to replace the molybdenum. It is considered that molybdenum(IV) oxide, generated on the metal surface from carbon dioxide or contaminating gaseous water or oxygen, also accomplishes the reduction. This may serve to underpin the reliability of this method. In a comparative study, a molybdenum column, although needing to be heated to a much higher temperature, required less and easier maintenance than a zinc column, and produced acceptably high and more reproducible yields (up to 71%) [36]. Therefore, this method has been widely adopted [31,39,40].

Notwithstanding these advances, another method for producing [^11^C]carbon monoxide from [^11^C]carbon dioxide has been proposed. This method is based on the decomposition of carbon dioxide at room temperature and atmospheric pressure by non-thermal dielectric barrage discharge [41]. The proof of principle for this method was shown with small macro amounts of non-radioactive carbon dioxide. The generated carbon monoxide was directly used as it was formed in microfluidic carbonylation reactions. However, this proposed method has not been adapted and demonstrated with NCA [^11^C]carbon dioxide.

#### 3.1.3. Into [^11^C]Hydrogen Cyanide

Niisawa et al. [42] produced about 200 mCi of [^11^C]hydrogen cyanide with a molar activity of 198 mCi/μmol by microwave discharge through a nitrogen–hydrogen-[^11^C]carbon dioxide mixture producing 40% yield within 20 min of ERP (Method a, Scheme 3C). Hara and Iio [43] produced [^11^C]hydrogen cyanide from [^11^C]carbon dioxide (150 to 160 mCi) that had been generated from a low-dose irradiation of nitrogen (9.4 MeV protons at 10 μA for 30 min). [^11^C]carbon dioxide was mixed with hydrogen and ammonia and passed over platinum at 950 °C, producing [^11^C]hydrogen cyanide in almost quantitative yield (Method b, Scheme 3C). These methods have not been adapted to high-level production. 

#### 3.1.4. Into [^11^C]Carbon Disulfide

Niisawa et al. [42] used microwave discharge on [^11^C]carbon dioxide with hydrogen sulfide and argon to produce 56 mCi of [^11^C]carbon disulfide in 9% yield with a molar activity of 42 mCi/μmol within 14 min from ERP (Scheme 3D). 

### 3.2. Conversions of [^11^C]Methane

#### 3.2.1. Into [^11^C]Carbon Dioxide

**Scheme 4:**
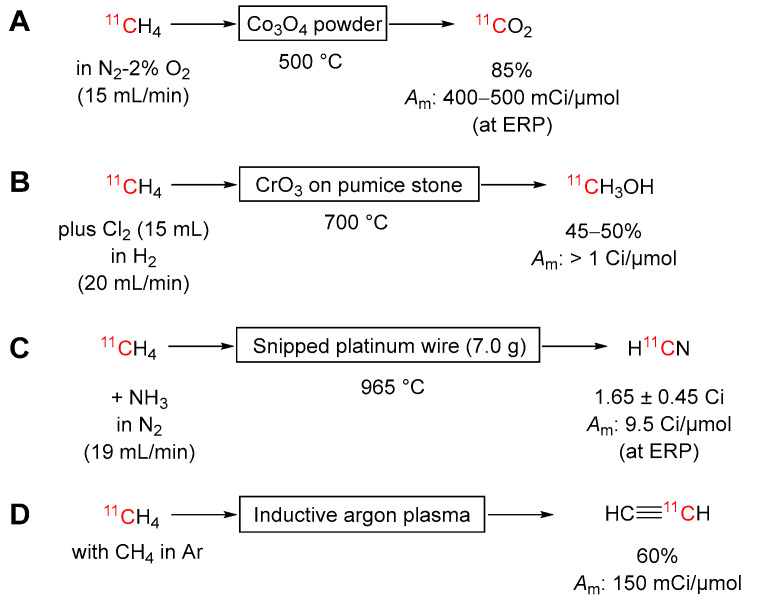
Conversion of [^11^C]methane into (**A**) [^11^C]carbon dioxide; (**B**) [^11^C]methanol; (**C**) [^11^C]hydrogen cyanide; and (**D**) [^11^C]acetylene.

[^11^C]Methane is easily converted into [^11^C]carbon dioxide by passage over cobalt(II-III) oxide (Co_3_O_4_) powder heated to 500 °C in a stream of nitrogen containing oxygen (2%) (Scheme 4A) [44]. The yield is 85%, but 60% isotopic dilution occurs, giving a low molar activity (400 to 500 mCi/μmol, decay corrected to ERP in the original study). This process does allow a single cyclotron target to be used as a source of both [^11^C]methane and [^11^C]carbon dioxide. 

#### 3.2.2. Into [^11^C]Methanol

Cyclotron-produced [^11^C]methane has been converted into [^11^C]methanol in 45 to 50% yield by passage with chlorine (15 mL) in hydrogen (20 mL/min) over heated chromium trioxide (CrO_3_) on pumice stone at 700 °C (Scheme 4B) [45]. Hydrogen was used to prevent oxidation to [^11^C]carbon monoxide or [^11^C]carbon dioxide, and chlorine was used to initiate radical formation. [^11^C]methanol was onward converted on-line into [^11^C]iodomethane (see Section 3.3.5) within 8 min from ERP, and then used to produce a tracer, [^11^C]flumazenil, with a molar activity of 1 Ci/μmol.

#### 3.2.3. Into [^11^C]Hydrogen Cyanide

[^11^C]Methane is quantitatively converted into [^11^C]hydrogen cyanide by passage with ammonia over platinum at 1000 °C [9,46,47]. Ample ammonia for this conversion is produced within a nitrogen–hydrogen gas target under an intense proton irradiation for [^11^C]methane production [9]. If [^11^C]methane is produced by the reduction of cyclotron-produced [^11^C]carbon dioxide, ammonia needs to be added to [^11^C]methane. Iwata et al. [48] achieved over 95% yield by passing [^11^C]methane with ammonia (5 vol %) in carrier nitrogen (200 mL/min) over platinum wire (0.2 mm o.d × 2 cm; 5.4 g) at 920 °C. The prior removal of oxygen and water impurities in the [^11^C]methane feed with Oxisorb gave an optimal yield of [^11^C]hydrogen cyanide. This method underpins commercially available ‘boxes’ for enacting this conversion [49,50]. Labaree et al. slightly modified the column configuration housing the platinum catalyst in the commercial device and achieved increased production yields of 1.65 ± 0.45 curies (*n* = 73) over a two-year period (Scheme 4C) [50]. With this source of [^11^C]hydrogen cyanide, they labeled a compound with a molar activity of 9.5 Ci/μmol (corrected to ERP). 

The applications of [^11^C]hydrogen cyanide to tracer synthesis are extensive [49,51] and mainly achieved through the nucleophilicity of the [^11^C]cyanide ion that can be generated from trapped hydrogen cyanide in a basic solution.

#### 3.2.4. Into CA [1-^11^C]Acetylene

The pyrolysis of [^11^C]methane with added carrier methane in an inductive argon plasma has produced [^11^C]acetylene in 60% yield within 10 min (Scheme 4D) [52]. Only a very low yield was obtained in the absence of an added carrier. Notably, carrier-added [^11^C]acetylene, that was produced by the proton irradiation of calcium carbide followed by hydrolysis, earlier found application for labeling two steroids, 17α-ethynylestradiol [53] and moxestrol [54].

#### 3.2.5. Into [^11^C]Fluoroform

**Scheme 5:**
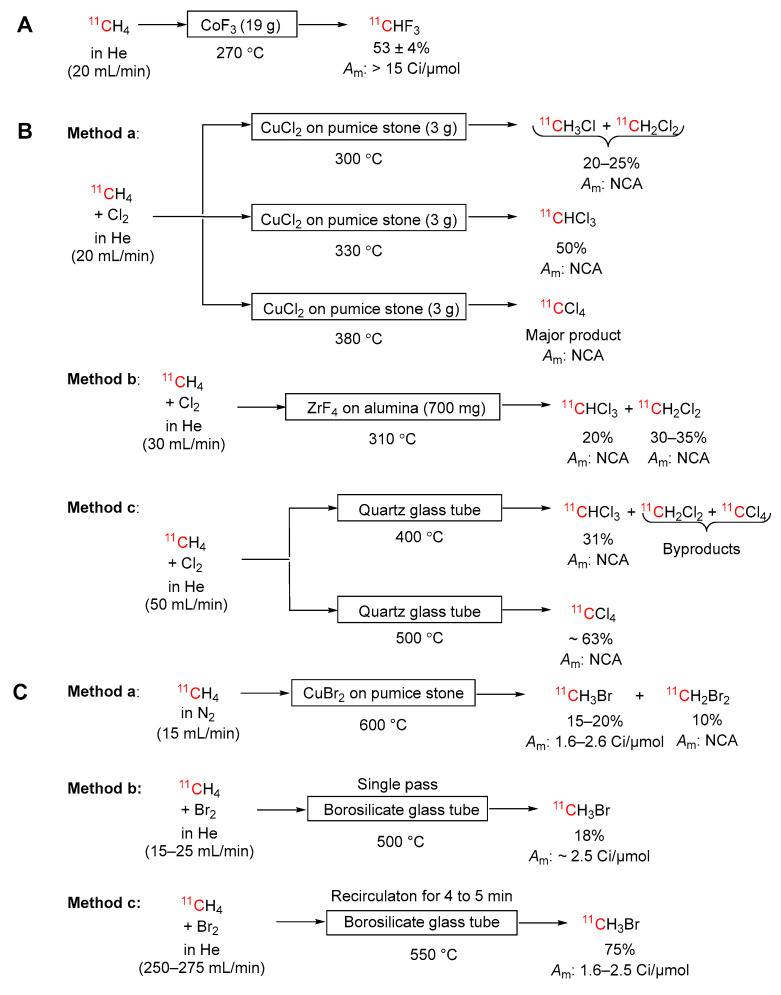

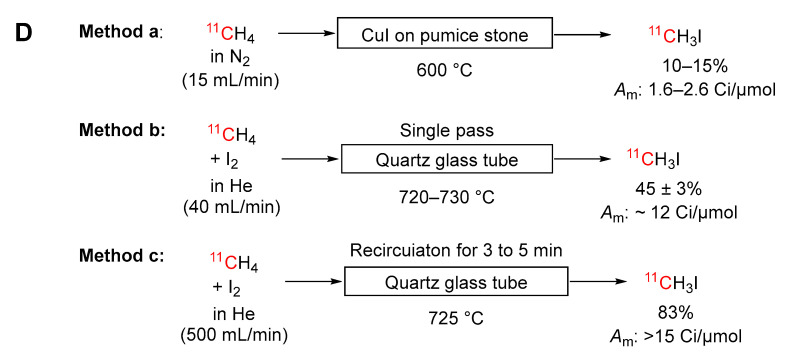
Halogenations of [^11^C]methane to produce useful labeling synthons: (**A**) fluorination; (**B**) chlorination; (**C**) bromination; and (**D**) iodination.

[^11^C]Methane is readily converted into [^11^C]fluoroform in 53 ± 4% yield (*n* = 11) by passage in a stream of helium (20 mL/min) over cobalt(III) fluoride (19 g) in a stainless steel tube at 270 °C (Scheme 5A) [55]. The whole process takes only 7 min from ERP. A single column was shown to operate consistently for over 80 runs without a need to change the cobalt(III) fluoride. [^11^C]Fluoroform so formed is readily converted into its reactive copper(I) derivative ([^11^C]CuCF_3_), which has a rich and developing chemistry for introducing [^11^C]trifluoromethyl groups into organic compounds [56,57,58,59]. Such compounds could be labeled with high molar activity (15 Ci/μmol). Ramos-Torres et al. [58] modified the procedure to increase the contact time between [^11^C]fluoroform and the cobalt(III) fluoride. This increased the purity of the [^11^C]fluoroform effluent from 88 to about 94%. [^11^C]Fluoromethane and [^11^C]difluoromethane are the main byproducts. 

#### 3.2.6. Into [^11^C]Chloromethanes

The passage of purified and concentrated [^11^C]methane with chlorine in helium over copper(II) chloride on pumice stone (3 g) produces [^11^C]chloroform at 330 °C in 45 to 50% yield (Method a, Scheme 5B) [60]. [^11^C]Chloroform prepared in this manner has been used to prepare [^11^C]diazomethane in solution [60]. At higher temperature (380 °C), the same catalytic system converts [^11^C]methane mainly into [^11^C]carbon tetrachloride. This chlorination is an important first step in a method for preparing [^11^C]phosgene [15] (see Section 3.3.7). Below 300 °C, [^11^C]chloromethane and [^11^C]dichloromethane are produced in moderate yields (20–25%) [61]. Crouzel and Hinnen [61] also explored other catalysts. In a meeting abstract, they reported 30 to 35% yield for [^11^C]dichloromethane (accompanied by 20% yield of [^11^C]chloroform) by the use of 10% zirconium tetrafluoride on alumina (700 mg) at 310 °C with a helium carrier gas flow rate of 30 mL/min (including added chlorine gas) (Method b, Scheme 5B). The ability of [^11^C]dichloromethane to effect ring closure on catechol was demonstrated.

Later, Solbach and Machulla [62] showed that [^11^C]methane could be chlorinated without catalyst (Method c, Scheme 5B). Using helium at 50 mL/min to carry a mixture of [^11^C]methane and chlorine through a quartz glass column at 400 °C, [^11^C]chloroform was obtained in 31% yield with [^11^C]dichloromethane and [^11^C]tetrachloromethane as byproducts. [^11^C]Chloroform from this method is also readily converted into [^11^C]diazomethane [62]. High-temperature chlorination (500 °C) predominantly produces [^11^C]tetrachloromethane (~63%). 

#### 3.2.7. Into [^11^C]Bromomethane 

Prenant and Crouzel passed [^11^C]methane over copper(II) bromide–pumice stone at 600 °C and produced [^11^C]bromomethane and [^11^C]dibromomethane in 15–20% and 10% yields, respectively (Method a, Scheme 5C) [63]. Mock et al. [64] showed that [^11^C]methane may be converted into [^11^C]bromomethane by reaction with heated bromine gas without catalyst. They performed this conversion in two modes: (i) by single pass through a tube reactor (Method b, Scheme 5C) and (ii) by rapid recirculation of [^11^C]methane through a similar tube reactor (Method c. Scheme 5C). In single pass mode, [^11^C]methane is passed with bromine though a narrow borosilicate glass column (2 mm i.d.) held at 500 °C, giving 18% conversion into [^11^C]bromomethane. These results questioned whether the copper(II) bromide–pumice stone was functional in the Prenant and Crouzel method [63]. Mock et al. introduced the recirculation of [^11^C]methane with the aim of enhancing the overall conversion into [^11^C]bromomethane. A carbon molecular sieve trap at room temperature was placed in the recirculation path to trap and accumulate the generated [^11^C]bromomethane. The optimal temperature for bromination was found to be between 525 and 550 °C, with transit times of 0.2 s at 250 mL/min, producing 70 to 75% [^11^C]bromomethane after only 4 to 5 min of recirculation. The relatively stable and highly volatile [^11^C]bromomethane (bp, 3.56 °C) can be efficiently recovered and carried over considerable distances through thin tubing for use as a ^11^C-methylation agent [64] for the on-line synthesis of the more reactive [^11^C]iodomethane (see Section 3.3.10) or [^11^C]methyl triflate (see Section 3.3.9). 

#### 3.2.8. Into [^11^C]Iodomethane

For several decades, [^11^C]iodomethane has been the most widely used ^11^C-labeling agent. This is mainly because of its reactivity towards heteroatom nucleophiles, such as amino groups, phenoxides, amides, and thiolates, thereby allowing the introduction of a ^11^C-methyl group into a broad swathe of potential PET tracers. For a long period, starting in the late 1970s, [^11^C]iodomethane was routinely produced by the reduction of [^11^C]carbon dioxide to [^11^C]methanol followed by iodination with hydroiodic acid [65]. However, such methods were not so ‘user friendly’ because of the need to use vulnerable and aggressive reagents (i.e., lithium aluminum hydride and hydroiodic acid) and the complexity of the required automation. For such reasons, various PET centers explored the direct gas phase iodination of [^11^C]methane for [^11^C]iodomethane synthesis (Scheme 5D) [25,66]. 

Prenant and Crouzel [63] reported a 10 to 15% yield of [^11^C]iodomethane by passing [^11^C]methane in nitrogen (15 mL/min) over copper(I) iodide on pumice stone at 600 °C (Method a, Scheme 5D). Later, Link et al. studied the reaction of [^11^C]methane with iodine vapor [66]. Iodine was heated to a high temperature (720 to 730 °C) in a quartz tube to generate free iodine radicals. These could extract hydrogen from [^11^C]methane in a stream of helium (35 to 40 mL/min) to produce [^11^C]methyl radicals that are capable of reacting with iodine (I_2_) to produce [^11^C]iodomethane. [^11^C]Iodomethane was obtained in more than 45 ± 3% yield (*n* = 7) with a molar activity of 12 Ci/μmol after only 4 min (Method b, Scheme 5D) [25]. The synthesis could be repeated without any apparatus clean-up. Zhang and Suzuki [22] investigated the sources of carrier in this single pass method. They suggested that traces of organic solvents and non-volatile oils (e.g., from fingerprints) could react with iodine vapor to yield carrier iodomethane in the heated quartz tube. Therefore, great care is needed to avoid such contamination. 

Larsen et al. developed an apparatus for recirculating unreacted [^11^C]methane in helium (~500 mL/min) through iodine vapor at 725 °C to produce [^11^C]iodomethane continuously. [^11^C]iodomethane was separated from the reactants on each cycle by entrapment on Porapak N, from which it could be later released at 190 °C in helium, either to a solution reaction medium or to a ‘loop reactor’ [67]. Under optimal conditions, [^11^C]iodomethane was obtained in a high yield (83%) with a molar activity exceeding 15 Ci/μmol at 7 min after collecting [^11^C]methane [24,66]. The simplicity and reliability of this method has led to its widespread use. Automated synthesizers that have been produced and sold by GE Healthcare, such as the TRACERlab FX C Pro in 2008 and the TRACERlab FX MeI in 2010, incorporate this method for [^11^C]iodomethane synthesis [68]. These devices can reliably produce [^11^C]iodomethane in 50 to 55% yields from either [^11^C]carbon dioxide or [^11^C]methane feeds in about 14 or 10.5 min from ERP, respectively. They have the facility to convert cyclotron-produced [^11^C]carbon dioxide to [^11^C]methane, as described in Section 3.1.1.

Synthra GmBH also produces an automated synthesizer for [^11^C]iodomethane synthesis from [^11^C]methane, which does not use recirculation but instead a series of three iodination reactors. This device produces about 50% yield within 8 min from ERP [68]. 

### 3.3. Gas Phase Transformations of Other ^11^C-Labeled Compounds

#### 3.3.1. [^11^C]Carbon Monoxide into [^11^C]Phosgene

For several decades, [^11^C]phosgene has been an important labeling synthon for introducing [^11^C]carbonyl groups into PET tracers [69]. However, the production of [^11^C]phosgene in high molar activity and yield has been challenging, giving rise to many diverse apparatuses and methods [69]. 

**Scheme 6:**
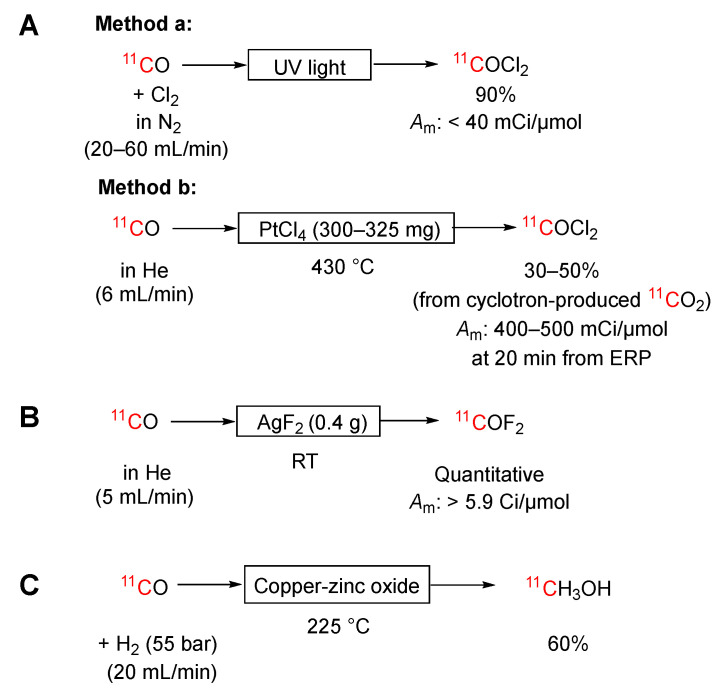
Conversions of [^11^C]carbon monoxide into other labeling synthons and intermediates: (**A**) [^11^C]phosgene; (**B**) [^11^C]carbonyl difluoride; and (**C**) proposed for [^11^C]methanol.

[^11^C]Carbon monoxide served as an intermediate in the early gas phase routes to [^11^C]phosgene. The first of these routes used ultraviolet light to promote the reaction of [^11^C]carbon monoxide with chlorine, producing [^11^C]phosgene in 75 to 90% yield (Method a, Scheme 6A) [70,71]. The second used platinum tetrachloride to achieve the same reaction (Method b, Scheme 6A) [72,73]. A 30-minute irradiation with a 30 μA proton beam consistently produced 200 to 300 mCi of [^11^C]phosgene. However, the molar activity achieved with either method was very low due to the carbon monoxide contamination of the chlorine in the first method (40 mCi/μmol) [71] and of platinum tetrachloride in the second (400 to 500 mCi/μmol at 20 min from ERP) [73]. These methods were quickly superseded by methods from [^11^C]methane via [^11^C]carbon tetrachloride [15] (see Section 3.3.7).

#### 3.3.2. [^11^C]Carbon Monoxide into [^11^C]Carbonyl Difluoride 

The passage of [^11^C]carbon monoxide in helium (5 mL/min) over a column of silver(II) fluoride (0.4 g) at room temperature provides [^11^C]carbonyl difluoride (bp, −85 °C) in quantitative yield within 7 min of ERP (Scheme 6B) [74]. This product is readily trapped in organic solvents and has been shown to label a broad range of cyclic substrates, for example, imidazolidin-2-ones, thiazolidin-2-ones, and oxazolidin-2-ones [74], and unsymmetrical acyclic ureas [75]. High molar activity (>5.9 ± 1.6 Ci/μmol at end of synthesis) was measured on a labeled product obtained from [^11^C]carbonyl fluoride. A silver(II) fluoride column could be used reliably for at least ten runs. The reactivity of [^11^C]carbonyl difluoride is very similar to the more difficult to produce [^11^C]phosgene and may be set to supersede the latter in many applications. 

#### 3.3.3. [^11^C]Carbon Monoxide into [^11^C]Methanol

A reactor has been designed to allow trace amounts of carbon monoxide to react with hydrogen to form methanol on a copper–zinc oxide catalyst [76]. Reduced and passivated copper–zinc oxide catalyzes the reaction of 50 ppm carbon monoxide with hydrogen to form methanol at 180–240 °C and 798 psi, and with a gas flow of 26–935 mL/min. A kinetic model was fitted to the experimental data with a commercially available process simulator (Hysys). Because the methanol synthesis is exothermic, as the temperature increases, the equilibrium conversion of carbon monoxide to methanol decreases. Also, for a given temperature, as the pressure increases, the equilibrium conversion of carbon monoxide to methanol increases. At 700 psi, the equilibrium conversion of carbon monoxide to methanol is quantitative below 180 °C and decreases to 90% at 240 °C. 

The model predicted optimal operating conditions for practical quantities of [^11^C]methanol. It was predicted that over 60% conversion of [^11^C]carbon monoxide into [^11^C]methanol could be achieved at 224 °C and 798 psi with a gas flow of 20 mL/min (Scheme 6C). The proposed reactor was considered suitable for incorporation into a process that might require [^11^C]methanol, such as the production of [^11^C]iodomethane with a high molar activity. The experimental results indicated that carbon monoxide and/or carbon dioxide can adsorb on the copper–zinc oxide surface at ambient temperature but desorb at about 200 °C. Accordingly, care would need to be taken to remove any traces of carbon dioxide or carbon monoxide before the use of the catalyst to ensure a high molar activity for the final [^11^C]methanol. This might be accomplished by flushing the reactor with pure helium before use, with the reactor heated slightly above the operating temperature. The reactor would also need to remain under inert gas while not in use, preferably slightly under pressure, to avoid contamination. The advantage of the proposed gas phase synthesis over the traditional liquid phase method [77] would be the potential to obtain ^11^C-labeled tracers in a high molar activity. However, there is yet no report for the implementation of this proposal, probably because of the requirement of hydrogen to be at high pressure.

#### 3.3.4. [^11^C]Methanol into [^11^C]Formaldehyde

**Scheme 7:**
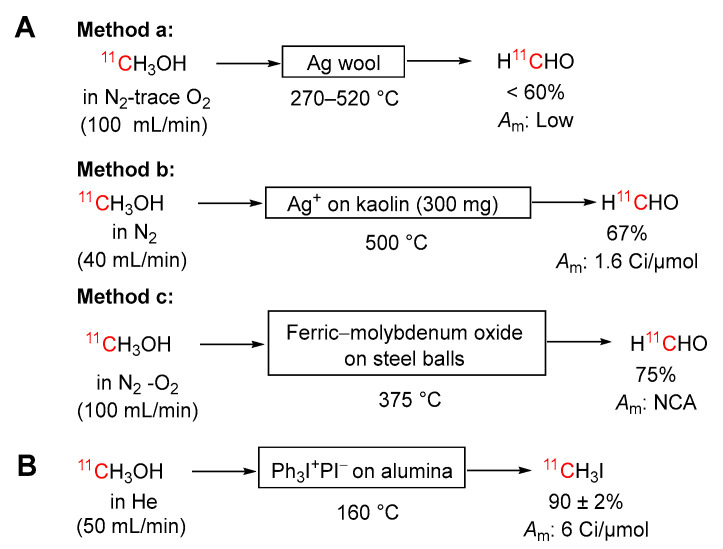
Conversions of [^11^C]methanol into (**A**) [^11^C]formaldehyde and (**B**) [^11^C]iodomethane.

[^11^C]Formaldehyde can be produced through silver wool-catalyzed high temperature (270–520 °C) oxidation of [^11^C]methanol in nitrogen containing a low percentage of oxygen (Method a, Scheme 7A) [77,78,79]. However, the yield depends strongly on the activity of the catalyst, is poorly reproducible, and seldom exceeds 60%. The catalytic activity of the silver depends on its prior use. With only a low concentration of oxygen in the carrier gas (ppm levels), the catalyst gradually deactivates. At a higher oxygen concentration (e.g., 2%), the catalyst increases in activity, tending to further oxidize [^11^C]methanol. For optimal molar activity, it is important to prevent non-radioactive contaminants in [^11^C]methanol from accessing the silver catalyst, for example, by passing [^11^C]methanol through a Porapak P (60–80 mesh) trap [80]. Pretreatment of the catalyst with methanol at 370 °C increases yield but reduces molar activity. More recently, Roeda and Dollé [81] described the use of a Ag^+^ ion-containing kaolin catalyst (300 mg; 20% silver content) in a 5 mm long column in place of silver wool to achieve the oxidation of [^11^C]methanol at 500 °C (Method b, Scheme 7A). This method produced [^11^C]formaldehyde in 67% yield with a molar activity up to 1.6 Ci/μmol.

As an alternative to silver or silver ion catalysis, [^11^C]methanol may be oxidized to [^11^C]formaldehyde in about 70% yield by passage with oxygen over a ferric–molybdenum oxide preparation supported on stainless steel balls at 375 °C (Method c, Scheme 7A) [78,82]. The production of [^11^C]formaldehyde requires about 10 min. 

As discussed above, there is no practical on-line gas phase method for producing the starting [^11^C]methanol for these methods. The required [^11^C]methanol may be produced from cyclotron-produced [^11^C]carbon dioxide by passage into lithium aluminum hydride in tetrahydrofuran followed by hydrolysis [79]. Even with the need for this prior step, [^11^C]formaldehyde can normally be produced in about 10 min. Molar activities up to 1 Ci/μmol have been reported [79].

[^11^C]Formaldehyde finds utility mainly for the ^11^C-methylation of secondary amines by reductive alkylation, such as in the synthesis of [^11^C]erythromycin A [76].

#### 3.3.5. [^11^C]Methanol into [^11^C]Iodomethane

Holschbach and Schuller produced [^11^C]iodomethane in amounts of up to 800 mCi by passage of [^11^C]methanol in a stream of helium (50 mL/min) over alumina-supported triphenylphosphine diiodide (1 g) held within a glass column at 160 °C. [^11^C]iodomethane was obtained in 90 ± 2% yield (*n*= 25) with a radiochemical purity of >99.5% and a mean molar activity of 6 Ci/μmol within an overall synthesis time of 11 min (Scheme 7B) [83]. [^11^C]methanol was produced by ‘wet’ reduction of cyclotron-produced [^11^C]carbon dioxide with lithium aluminum hydride. Fournier and Crouzel used this type of procedure to produce [^11^C]iodomethane from [^11^C]methanol that had been produced on-line from [^11^C]methane (see Section 3.2.2) [45]. 

#### 3.3.6. [^11^C]Ethanol into [1-^11^C]Ethylene 

**Scheme 8:**
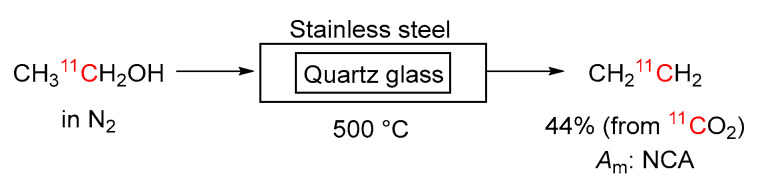
Conversion of [1-^11^C]ethanol into [1-^11^C]ethylene.

[1-^11^C]Ethylene may be prepared by passing [1-^11^C]ethanol over quartz glass held entirely within a heated (500 °C) stainless steel tube (Scheme 8) [84]. [1-^11^C]ethanol is ideally prepared from cyclotron-produced [^11^C]carbon dioxide by ^11^C-carboxylation of methylmagnesium bromide that has been freshly prepared in dibutyl ether, followed by reduction of the generated adduct with lithium aluminum hydride in diglyme. The use of involatile solvents avoids the undesirable formation of carrier ethylene and radioactive and stable diethyl ether by trace solvent cracking over the heated catalyst. Placing the quartz glass entirely within the heated zone of the stainless steel tube avoided adsorptive radioactivity losses. This method was preferred to catalytic dehydration over alumina or pyrolysis at 700 °C. The radiosynthesis takes 21 min and has a radiochemical yield of 44%, from [^11^C]carbon dioxide. [1-^11^C]Ethylene may be converted quantitatively into [1-^11^C]l,2-dibromoethane when collected in a solution of bromine in carbon tetrachloride. The NCA [1-^l1^C]ethylene and [1-^11^C]l,2-dibromoethane have the potential to serve as new and useful 2-carbon labeling agents.

#### 3.3.7. [^11^C]Carbon Tetrachloride into [^11^C]Phosgene

**Scheme 9:**
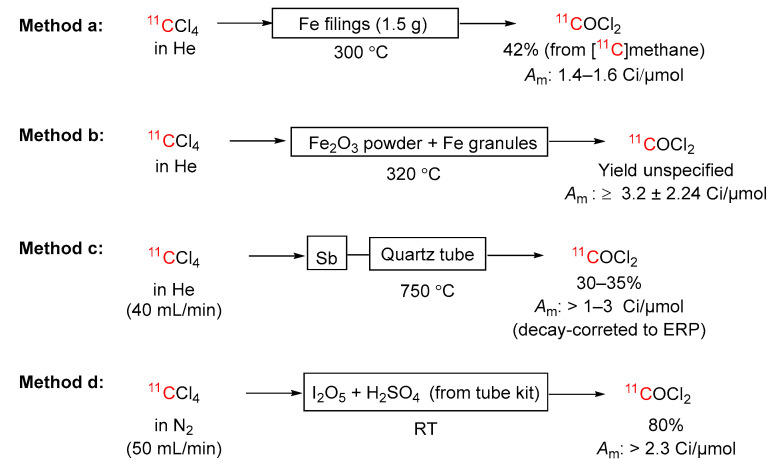
Conversions of [^11^C]carbon tetrachloride into [^11^C]phosgene.

[^11^C]Phosgene may be produced from [^11^C]carbon tetrachloride by passage in helium over iron filings (1.5 g) at 300 °C (Method a, Scheme 9) [15]. Surface oxide on the filings presumably provides the required oxygen. The generated [^11^C]phosgene is passed through a trap (3 mm i.d.) of antimony (400 mg) mixed with glass beads (1 mm o.d.) to remove traces of chlorine before collection in a desired solvent. This conversion normally follows the on-line production of [^11^C]carbon tetrachloride from cyclotron-produced [^11^C]methane, as described earlier (Section 3.2.5). The coupled two-step process takes about 10 to 12 min. A 30-minute cyclotron irradiation with a 30 μA beam of 20 MeV protons was reported to produce 375 to 500 mCi of [^11^C]phosgene with a molar activity of 1.4 to 1.6 Ci/μmol, corresponding to 2.5 carrier dilution with respect to the molar activity of starting [^11^C]methane.

Nishijima et al. [85] sought to improve the synthesis of [^11^C]phosgene from [^11^C]carbon tetrachloride. They examined two oxidizing agents, iron(III) oxide and copper(II) oxide. The yield of [^11^C]phosgene, based on labeled derivatives, was significantly increased using iron(III) oxide powder mixed with iron granules, whereas the use of copper(II) oxide alone, or copper(II) oxide powder mixed with iron granules, resulted in insignificant yield. The yield and molar activity of the β-adrenoceptor radioligand, [^11^C](*S*)-CGP-12177, from [^11^C]phosgene that was prepared using iron(III) oxide powder mixed with iron granules (Method b, Scheme 9), were markedly higher than by previous methods using iron granules alone or iron granules mixed with iron powder.

Bramoullé et al. described a simplified on-line method for the production of [^11^C]phosgene [86]. In this method, cyclotron-produced [^11^C]methane is mixed with chlorine and converted into [^11^C]carbon tetrachloride by passage through an empty quartz tube at 510 °C. The outflow is then directed through an antimony-filled guard column that removes chlorine and then, without intentional oxygen addition, through a second empty quartz tube at 750 °C, produces [^11^C]phosgene in 30 to 35% yield in a total radiosynthesis time of about 12 min (Method c, Scheme 9). 

Ogawa et al. [87] developed a room temperature method for synthesizing [^11^C]phosgene. This method is based on utilizing part of a carbon tetrachloride detection tube kit intended for environmental air analysis (Method d, Scheme 9). This used part is a pretreatment tube that is filled with a support material containing iodine pentoxide (I_2_O_5_) and fuming sulfuric acid. The optimal flow of [^11^C]carbon tetrachloride through the tube was 50 mL/min. [^11^C]Phosgene was obtained in 80.8 ± 3.1% yield (*n* = 5) from [^11^C]carbon tetrachloride after 10 min of total radiosynthesis time and produced a molar activity of 2.3 ± 1.4 Ci/μmol (*n* = 3) on a labeled derivative. The synthesis apparatus was automated for preparing [^11^C]phosgene up to 4 times per week [88].

#### 3.3.8. [^11^C]Iodomethane into [^11^C]Methyl Triflate

**Scheme 10:**
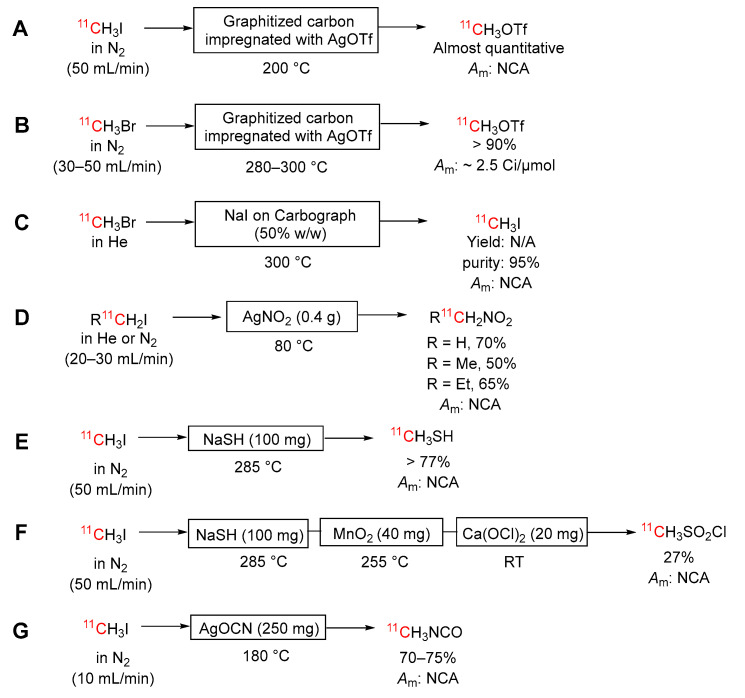
Metathetical conversions of [^11^C]haloalkanes into useful labeling synthons by passage over heated silver(I) or sodium salts into (**A**,**B**) [^11^C]methyl triflate; (**C**) [^11^C]iodomethane; (**D**) [^11^C]nitroalkanes; **(E**) [^11^C]methanethiol; (**F**) [^11^C]mesyl chloride; and (**G**) [*methyl*-^11^C]methyl isocyanate.

[^11^C]Methyl triflate is a more powerful methylation agent than [^11^C]iodomethane [89,90]. This labeling agent can be prepared in almost quantitative yield and within 7 min simply by passing [^11^C]iodomethane in a stream of nitrogen gas (30–100 mL/min) over graphitized carbon (~600 mg) impregnated with 50% by weight of silver(I) triflate held within a small Pyrex tube (6 mm o.d. × 23 cm) at 150–200 °C (Scheme 10A) [91]. [^11^C]methyl triflate (bp, 100 °C) is readily trapped in organic solvents at room temperature. The molar activity of [^11^C]methyl triflate is dictated by the molar activity of the [^11^C]iodomethane feed; the process does not add carrier. The very extensive utility that [^11^C]methyl triflate has gained in the PET radiochemistry field for labeling tracers at heteroatoms has been recently summarized [92].

#### 3.3.9. [^11^C]Bromomethane into [^11^C]Methyl triflate

[^11^C]Bromomethane may be converted into [^11^C]methyl triflate in a similar manner to [^11^C]iodomethane (Scheme 10B) [64]. Thus, passage of [^11^C]bromomethane in a helium stream (30–50 mL/min) through a column of graphitized carbon impregnated with silver(I) triflate held between 280 and 300 °C produces [^11^C]methyl triflate in over 90% yield. Compounds labeled with [^11^C]methyl triflate from this method have shown molar activities of about 2.5 Ci/μmol. This method is incorporated into an automated synthesis device, called Modular Lab, offered commercially by Eckert & Ziegler Radiopharma GmbH [68]. 

#### 3.3.10. [^11^C]Bromomethane into [^11^C]Iodomethane

The passage of [^11^C]bromomethane through a column of sodium iodide on Carbograph at 300 °C produces [^11^C]iodomethane in 95% purity, although the yield was not reported (Scheme 10C). This chemistry is also incorporated into the automated synthesis device (Modular Lab) offered commercially by Eckert & Ziegler [68]. 

#### 3.3.11. Lower [1-^11^C]Iodoalkanes into Lower [1-^11^C]Nitroalkanes

[^11^C]Nitromethane can be prepared simply and efficiently by passing [^11^C]iodomethane in nitrogen or helium at 20 to 30 mL/min through a soda glass column (3 mm i.d. × 4 cm) packed with silver(I) nitrite (0.4 g) at 80 °C (Scheme 10D) [93]. Higher [^11^C]nitroalkanes can be prepared similarly from volatile [1-^11^C]iodoalkanes (R^11^CH_2_I, R = Me, Et) [93], produced as described by Långström et al. [94]. [1-^11^C]nitroalkanes are readily trapped in solvents of choice for further reaction (e.g., dimethyl sulfoxide). The yields are 70, 50, and 65% for R = H, Me, and Et, respectively, based on the corresponding [1-^11^C]alkyl iodides and 55, 30 and 40% overall from starting [^11^C]carbon dioxide, in radiosynthesis times of 8 to 15 min. The on-line method for producing [1-^11^C]nitromethane overcame many limitations of the earlier solution method based on direct reaction with silver nitrite in an organic solvent at 80 °C [95], such as handling difficulties, poor reproducibility, and the limitations on solvents for further reactions. The heated sodium nitrite can produce nitrogen oxides, which can interfere in some labeling reactions containing [^11^C]nitromethane. However, the volatile [^11^C]nitromethane product can be purified on-line by placing a sodium carbonate plug in the heated column after the sodium nitrite [96]. The helium–[^11^C]nitromethane should then be dried by passage through Sicapent. [^11^C]Nitromethane reacts readily with aldehydes and has found early application for labeling D-glucose and D-mannose at their C-1 positions [97]. 

#### 3.3.12. [^11^C]Iodomethane into [^11^C]Methanethiol and then [^11^C]Mesyl Chloride

[^11^C]Methanethiol is of interest for introducing [^11^C]thiomethyl groups into organic structures, such as the amino acid L-methionine. [^11^C]Alkanethiols have been prepared in solution from [1-^11^C]iodoalkanes and commercially available sodium hydrosulfide (NaSH) [98,99]. Thus, [^11^C]methanethiol may be prepared almost instantaneously and quantitatively by dispensing [^11^C]iodomethane into a solution of sodium hydrosulfide in anhydrous dimethylformamide. Because [^11^C]methanethiol is gaseous (bp, 6 °C), a simple nitrogen purge of the reaction mixture enables the transfer of this product to a collection vessel. However, when McCarron and Pike [100] tried to implement this method, only about 60% of the initial [^11^C]iodomethane radioactivity was successfully transferred over a period of 10 min. This experience prompted the development of an on-line process for the preparation (Scheme 10E). Passage of [^11^C]iodomethane in nitrogen (50 mL/min) over sodium hydrosulfide (100 mg) in a silica tube (2 mm i.d. × 30 cm) at 285 °C generated [^11^C]methanethiol, which was collected in chlorine-saturated water and instantly converted to [^11^C]mesyl chloride. The yield from [^11^C]iodomethane was 77%. 

Oxidative chlorination of [^11^C]methanethiol to [^11^C]mesyl chloride could be achieved on-line by passage over manganese(IV) oxide (40 mg) in a silica tube (2 mm i.d. × 30 cm) at 255 °C and then over a short plug of calcium hypochlorite (20 mg) at room temperature (Scheme 10F). [^11^C]Mesyl chloride has potential as a labeling agent, as was shown by its ability to mesylate tetrahydroisoquinoline [100]. 

These on-line procedures are attractive for their ease of future radiosynthesis automation and operation with high radioactivity levels but require further optimization. 

#### 3.3.13. [^11^C]Iodomethane into [*methyl*-^11^C]Methyl Isocyanate 

[*methyl*-^11^C]Methyl isocyanate has been prepared by passing [^11^C]iodomethane in nitrogen (10 mL/min) for 1 minute over silver(I) cyanate (250 mg) at 180 °C (Scheme 10G) [101]. Typically, the conversion of [^11^C]iodomethane to [*methyl-*^11^C]methyl isocyanate was 70–75% with a total radiosynthesis time of 13 min, including 12 min for [^11^C]iodomethane production. About 25% of the radioactivity was retained on the silver(I) cyanate column. Higher temperature failed to drive off this radioactivity. Notably, [*methyl*-^11^C]methyl isocyanate was used to label the antitumor drug temozolomide in an *N*-methyl group [101].

#### 3.3.14. [^11^C]Iodomethane into [^11^C]Hydrogen Cyanide via [^11^C]Formaldehyde

**Scheme 11:**
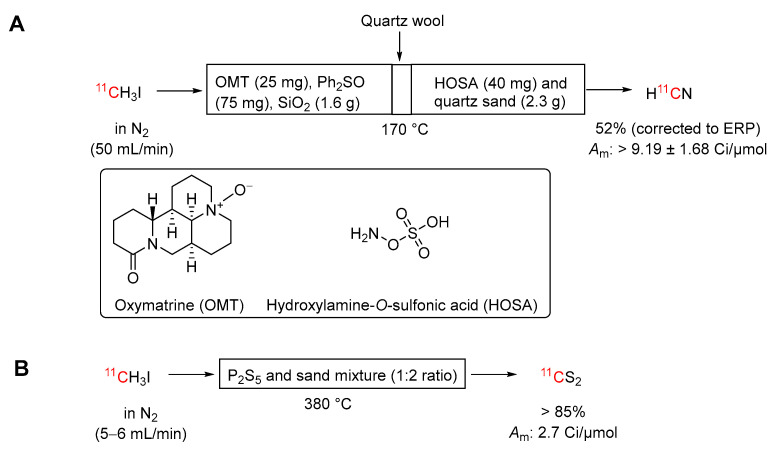
Conversions of [^11^C]iodomethane into (**A**) [^11^C]hydrogen cyanide and (**B**) [^11^C]carbon disulfide.

Kikuchi et al. [102] have described the conversion of [^11^C]iodomethane into [^11^C]hydrogen cyanide by passage in nitrogen through a small two-layered reaction column heated to 170 °C (Scheme 11A). The first layer contains a *N*-oxide (oxymatrine) and diphenyl sulfoxide for the conversion of [^11^C]iodomethane into [^11^C]formaldehyde. The generated [^11^C]formaldehyde is subsequently converted into [^11^C]hydrogen cyanide in a second layer containing hydroxylamine-*O*-sulfonic acid (HOSA). The yield of [^11^C]hydrogen cyanide from this method (~52%, corrected to ERP) is similar to that of [^11^C]hydrogen cyanide produced by the traditional method (51%). The molar activity of [^11^C]hydrogen cyanide, determined by derivatization, was estimated to be 9.43 Ci/μmol and almost the same as that of the starting [^11^C]iodomethane (9.32 Ci/μmol). The first layer of the column can be used to produce gaseous [^11^C]formaldehyde with a yield estimated from derivatization of about 83%. The authors provided a video on the assembly of the required bi-layer column. 

#### 3.3.15. [^11^C]Iodomethane into [^11^C]Carbon Disulfide 

[^11^C]Carbon disulfide (bp, 46 °C) has been produced by a gas phase reaction from [^11^C]iodomethane (Scheme 11B) [103]. In this procedure, a stream [^11^C]iodomethane is passed through a small glass column packed with a mixture of phosphorus pentasulfide and sand in 1: 2 ratio, at 380 °C. The gases are vented into a vial containing acetonitrile at room temperature, which conveniently traps [^11^C]carbon disulfide. The conversion of [^11^C]iodomethane into [^11^C]carbon disulfide is instantaneous, producing high yields (>85%) and high molar activities (2.7 Ci/μmol). The only major contaminant is a small amount of unreacted [^11^C]iodomethane. The flow rate of gas carrying [^11^C]iodomethane through the phosphorus pentasulfide column had a significant impact on conversions. A flow of 5–6 mL/min gave good conversions with an acceptable processing time of less than 10 min from the end of [^11^C]iodomethane production. Higher flow rates gave decreased yields. [^11^C]Carbon disulfide was reactive towards aniline, benzylamine, and diethylamine, thereby showing considerable potential for labeling prospective sulfur-containing tracers.

#### 3.3.16. [^11^C]Hydrogen Cyanide into [^11^C]Cyanogen Bromide

**Scheme 12:**
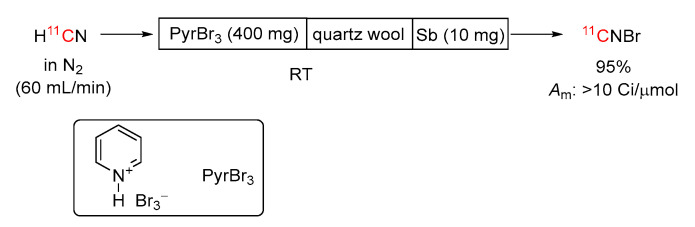
Conversion of [^11^C]hydrogen cyanide into [^11^C]cyanogen bromide.

[^11^C]Cyanogen bromide, an electrophilic labeling agent, has been produced in 95% yield from [^11^C]hydrogen cyanide within 3 min from ERP using a simple and convenient on-line procedure (Scheme 12) [104]. In this procedure, [^11^C]hydrogen cyanide is passed through a Sicapent tower (10 mm i.d. × 10 cm), and then through a quartz tube (6.25 mm i.d. × 15 cm) contacting first pyridinium perbromide (400 mg) and then antimony powder (10 mg) separated by a quartz wool plug. The generated [^11^C]cyanogen bromide can be trapped in a suitable solvent for further use. The molar activity of [^11^C]cyanogen bromide was difficult to determine directly and was therefore estimated from the molar activity of derivatives, producing values between 10 and 12 Ci/μmol at end of synthesis. The procedure has been automated [105]. This on-line method for [^11^C]cyanogen bromide synthesis is shorter, technically simple, and more reproducible than the earlier reported solution method [106]. [^11^C]Cyanogen bromide has been used to label several compounds, such as the antiviral compound GR121167X [107], albumin [108], and hyaluronic acid [109].

#### 3.3.17. [1-^11^C]Butyric Acid into [1-^11^C]Propylketene 

**Scheme 13:**
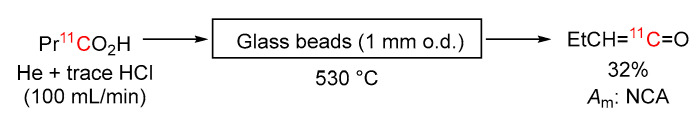
Conversion of [1-^11^C]butyric acid into [1-^11^C]propylketene.

Fujii et al. developed a method for preparing [1-^11^C]but-1-en-1-one ([1-^11^C]propylketene) in 32% yield based on the pyrolysis of [1-^11^C]butyric acid (bp, 164 °C) at 550 °C in a quartz tube (7 mm i.d. × 24 cm) containing glass beads (1 mm o.d.) [110]. [1-^11^C]butyic acid was prepared by ^11^C-carboxylation of propyl lithium with cyclotron-produced [^11^C]carbon dioxide followed by acidification with hydrogen chloride gas (Scheme 13). The [1-^11^C]butyric acid was carried through the quartz tube with helium (70 mL/min) containing a low concentration of dry hydrogen chloride. The whole radiosynthesis required 25 min giving an overall yield of 22%. Yields were measured after derivatization. Forskolin and several phorbol esters and diacylglycerols were successfully labeled with [1-^11^C]propylketene [111,112]. 

## 4. Conclusions and Outlook

Gas phase transformations play a major part in carbon-11 chemistry and in PET tracer utilization and development, largely because of their simplicity, reliability, re-usability, and amenability for automation. Major advances have been made through appropriate selection and optimization of reagents and catalysts and in the engineering of suitable reactors and their energy supplies. Further advances can be anticipated in all aspects.

## Figures and Tables

**Table 1:** Sections discussing ^11^C-labeled products from gas phase transformations.

Labeled Product	Formula	Section(s)
[^11^C]Acetylene	^11^CH≡CH	Section 3.2.4
[^11^C]Bromomethane	^11^CH_3_Br	Section 3.2.7
[^11^C]Carbon dioxide	^11^CO_2_	Section 3.2.1
[^11^C]Carbon disulfide	^11^CS_2_	Section 3.1.4 and Section 3.3.15
[^11^C]Carbon monoxide	^11^CO	Section 3.1.2
[^11^C]Carbonyl difluoride	^11^COF_2_	Section 3.3.2
[^11^C]Chloroform	^11^CHCl_3_	Section 3.2.6
[^11^C]Chloromethane	^11^CH_3_Cl	Section 3.2.6
[^11^C]Cyanogen bromide	^11^CNBr	Section 3.3.16
[^11^C]Dibromomethane	^11^CH_2_Br_2_	Section 3.2.7
[^11^C]Dichloromethane	^11^CH_2_Cl_2_	Section 3.2.6
[^11^C]Ethylene	^11^CH_2_CH_2_	Section 3.3.6
[^11^C]Fluoroform	^11^CHF_3_	Section 3.2.5
[^11^C]Formaldehyde	H^11^CHO	Section 3.3.4 and Section 3.3.14
[^11^C]Hydrogen cyanide	H^11^CN	Section 3.1.3, Section 3.2.3 and Section 3.3.14
[^11^C]Iodomethane	^11^CH_3_I	Section 3.2.8, Section 3.3.5 and Section 3.3.10
[^11^C]Mesyl chloride	^11^CH_3_SO_2_Cl	Section 3.3.12
[^11^C]Methane	^11^CH_4_	Section 3.1.1
[^11^C]Methanethiol	^11^CH_3_SH	Section 3.3.12
[^11^C]Methanol	^11^CH_3_OH	Section 3.2.2 and Section 3.3.3
[*methyl*-^11^C]Methyl isocyanate	^11^CH_3_NCO	Section 3.3.13
[^11^C]Methyl triflate	^11^CH_3_OTf	Section 3.3.8 and Section 3.3.9
[^11^C]Nitroethane	^11^CH_3_CH_2_NO_2_	Section 3.3.11
[^11^C]Nitromethane	^11^CH_3_NO_2_	Section 3.3.11
[^11^C]Nitropropane	^11^CH_3_CH_2_CH_2_NO_2_	Section 3.3.11
[^11^C]Phosgene	^11^COCl_2_	Section 3.3.1 and Section 3.3.7
[1-^11^C]Propylketene	EtCH=^11^C=O	Section 3.3.17

## Data Availability

All data used in this work were derived from publications. Summaries of extracted data are included in this work.
